# Effect of crystal plane orientation on tribochemical removal of monocrystalline silicon

**DOI:** 10.1038/srep40750

**Published:** 2017-01-13

**Authors:** Chen Xiao, Jian Guo, Peng Zhang, Cheng Chen, Lei Chen, Linmao Qian

**Affiliations:** 1 Tribology Research Institute, National Traction Power Laboratory, Southwest Jiaotong University, Chengdu 610031, Sichuan Province, P. R. China; 2 School of Mechatronics Engineering, University of Electronic Science and Technology of China, Chengdu 611731, Sichuan Province, P. R. China

## Abstract

The effect of crystal plane orientation on tribochemical removal of monocrystalline silicon was investigated using an atomic force microscope. Experimental results indicated that the tribochemical removal of silicon by SiO_2_ microsphere presented strong crystallography-induced anisotropy. Further analysis suggested that such anisotropic tribochemical removal of silicon was not dependent on the crystallography-dependent surface mechanical properties (i.e., hardness and elastic modulus), but was mainly attributed to various atomic planar density and interplanar spacing in different crystal planes. Phenomenological results speculated that higher density of silicon atom could promote the formation of Si-O-Si bonds between the SiO_2_ microsphere and silicon substrate, resulting in more severe tribochemical material removal. Larger interplanar spacing with smaller energy barrier facilitated the rupture of the Si-Si network with the help of mechanical shearing stress, which caused more serious wear of the silicon surface. The results may help understand the material removal mechanism of silicon and provide useful knowledge for chemical mechanical polishing.

Due to excellent electronic and mechanical properties, monocrystalline silicon has become the dominant substrate and structural material in integrated circuits and devices^1^,^2^,^3^,^4^. Monocrystalline silicon has three typical crystal planes, i.e., (100), (110) and (111). Based on anisotropic surface properties of silicon wafers with different plane orientations, these wafers were employed as substrate material in manufacturing various microelectronic products^5^. For example, Si(100) is used in complementary metal oxide semiconductor because of the lowest interfacial state and least fixed charge^6^. Mostly, bipolar transistors are inclined to choose Si(111) because of the close-packed plane^7^. In addition, Si(110) is often used as the substrate surface to grow low-dimensional structures such as nanowires^8^,^9^,^10^. Obtaining a planar, smooth and damage-free monocrystalline silicon surface for the aforementioned applications, requires understanding the material removal mechanism of silicon in chemical mechanical polishing (CMP) process^11^,^12^,^13^.

Generally, material removal is described by Archard law, which ascribes wear volume to the applied normal load and the material hardness^14^,^15^,^16^. However, numerous experimental results indicate that the material removal may not only depend on surface mechanical properties, but also on many intrinsic factors, such as bond energy and dangling-bond density^17^,^18^,^19^. Monocrystalline silicon is a typical anisotropic material, i.e., different crystal planes present different mechanical properties, such as elastic modulus, hardness and fracture toughness. Most of the previous studies focused on the anisotropic mechanical wear of silicon on various crystal planes^20^,^21^,^22^,^23^,^24^,^25^. For example, Stempflé *et al*. reported that the material removal of Si(100) was easier because the “softer” surface was more effective in producing cleavages^20^. By using nanoscratching tests, Gassilloud *et al*. presented that the micro cracks were likely to produce and expand across the cleavage plane (111) of silicon, which may lead to high wear rate of Si(100) surface^21^. However, no available literature was found to report the study on the anisotropic tribochemical removal of silicon.

In either the CMP process or the application of silicon-based microelectromechanical systems (MEMS), tribochemical removal played a more important role than the mechanical removal of silicon. Using an atomic force microscope (AFM), Yu *et al*. conducted wear tests between SiO_2_ microsphere and Si(100) substrate in humid air^19^. The results suggested that with the help of water molecules, Si-O-Si bonding bridges may form at Si/SiO_2_ interface and then induce the material removal. However, previous researches have mainly focused on Si(100), while the study on other crystal planes remains relatively scarce^17^,^18^,^19^,^20^,^21^,^22^,^23^,^24^,^25^,^26^. Based on the proposed tribochemical wear mechanism, the anisotropy in atomic density and interplanar spacing has great opportunities to affect the formation and rupture of Si-O-Si bonding bridges, and further induce the anisotropic tribochemical wear of silicon^15^,^19^,^27^. Therefore, clarification of the effect of crystal plane orientation is crucial for tribochemical removal of monocrystalline silicon.

In this paper, the nanowear of silicon surfaces with different crystal plane orientations was studied through AFM. The experimental results indicated that crystal plane orientation had a prominent effect on the tribochemical removal of silicon. The effects of mechanical property, atomic planar density and interplanar spacing on the anisotropic tribochemical removal of silicon were discussed. The investigation expands the understanding of the mechanism of silicon material removal and provides useful knowledge for chemical mechanical polishing of silicon wafers.

## Results

### Tribochemical wear of silicon by SiO_2_ spherical tip in water

**Figure 1:**
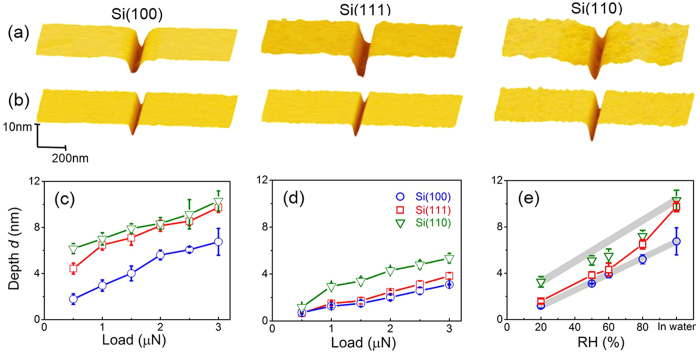
AFM observation of tribochemical wear on silicon surfaces by SiO_2_ tip. AFM images and cross-sectional profiles of nanowear tracks on Si(100), Si(110) and Si(111) by SiO_2_ microsphere in ultrapure water **(a)** and at 50% RH **(b)**, The applied normal load was 3 μN. Comparison of wear depths on silicon samples with different crystal planes under various normal loads in ultrapure water **(c)** and at 50% RH **(d)**. **(e)** Effect of relative humidity on the wear depths of silicon surfaces with different crystal planes. The applied normal load was 3 μN. The values of wear depths under the same loading conditions in water were also plotted as a comparison.

Tribochemical reaction played a crucial role in the material removal of silicon surface during the CMP process^28^. To investigate the effect of crystal plane orientation on tribochemical wear of silicon, nanowear tests on three typical silicon substrates were performed by spherical SiO_2_ tip in water. Figure 1a shows the AFM topographies and cross-sectional profiles of nanowear scars on Si(100), Si(110) and Si(111) surfaces. Obviously, the grooves formed on three silicon surfaces under all given normal loads. Figure 1c shows the wear depths on three silicon samples with different crystal planes at various normal loads. Wear scars produced on Si(110) were the deepest, while those produced on Si(100) were the shallowest. In addition, the wear depths on Si(111) were slightly smaller than those on Si(110) under the same loading condition. When the applied normal load *F*_n_ increased from 0.5 μN to 3 μN, the wear depth increased from 6.1 nm to 10.3 nm on Si(110), 4.4 nm to 9.5 nm on Si(111) and 1.8 nm to 6.7 nm on Si(100), respectively. These results indicated that tribochemical removal of silicon induced by SiO_2_ tip in water was strongly influenced by the crystal plane orientation. The involved mechanism is discussed at a later part of this paper.

### Tribochemical wear of silicon by SiO_2_ spherical tip in humid air

Clearly, the presence of water is the key factor in tribochemical wear behavior of Si/SiO_2_ pair. To further understand the crystallography-induced anisotropy in tribochemical wear of Si/SiO_2_ pair, nanowear tests were performed in humid air under the same experimental conditions. The similar material removal results were observed on three silicon surfaces in humid air, as shown in Fig. 1b and d. Compared to severe wear in water, the wear depths and widths in humid air have dramatically declined. In accordance with the case of nanowear experiments in water, Si(110) samples suffered the most severe wear, while the wear on Si(100) surface was the slightest. The wear depths of Si(100) and Si(111) in humid air were close. The friction coefficient of Si/SiO_2_ pair (see Figure S1 in Supplementary Information) showed almost no difference among the silicon samples with different crystal plane orientations either in humid air or in water. The results indicated that the anisotropic tribochemical removal of silicon was not attributed to the friction behavior of Si/SiO_2_ pair. With nanowear environment varying from humid air to water, the volume of adsorbed water in the contact area increased, which aggravated the wear of all silicon samples. The silicon surfaces with various crystal planes exhibited different increases in wear depth. For Si(100)/Si(110)/Si(111), the wear depth increased by 117%/112%/154%, respectively. Among all samples, the variation of Si(111) was the largest, while Si(100) and Si(110) had a similar variation rate. The result indicated that (i) the presence of water molecules in the surrounding environment played an important role in the tribochemical wear of silicon, and (ii) crystal plane orientation had a strong effect on humidity-dependent nanowear on the silicon surface.

### Effect of mechanical property on the anisotropic tribochemical wear of silicon

**Figure 2:**
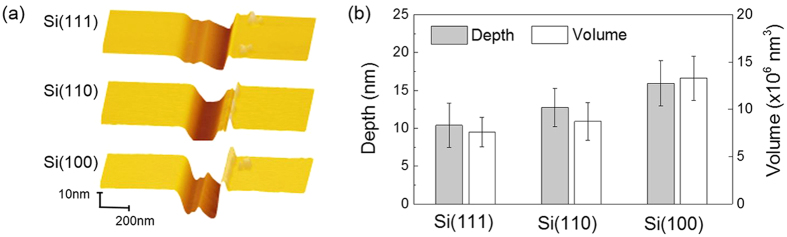
AFM observation of mechanical wear on silicon surfaces by diamond tip. **(a)** AFM images and cross-sectional profiles of nanowear tracks on Si(100), Si(110) and Si(111) by diamond tip. **(b)** Comparison of wear depths and volume on silicon surfaces with different crystal planes by diamond tip. The applied normal load was 50 μN.

To verify whether the anisotropic tribochemical wear of silicon was induced by the anisotropic mechanical property or not, nanowear tests were performed by a diamond tip with *R* ≈ 0.25 μm under *F*_n_ = 50 μN. Figure 2a shows AFM topographic images and cross-sectional profiles of wear scars on Si(100), Si(110) and Si(111) surfaces. Serious plastic deformation and material removal occurred on three silicon surfaces with different crystal planes. Wear debris and pileup of plastic deformation were observed around the worn area. Generally, the wear of Si/Diamond pair is dominated by mechanical wear, which is governed by the processes of deformation and fracture. Therefore, such wear behavior is strongly associated with surface mechanical properties^29^,^30^. Table 1 shows that the mechanical properties (hardness, elastic modulus) for the three crystal planes can be ranked by: Si(111) > Si(110) > Si(100)^24^,^29^. Figure 2b shows the comparison of wear depths and volume for the three crystal planes, that is, Si(111) < Si(110) < Si(100). The phenomenon was consistent with the measuring results by Bhushan *et al*.^22^. Due to the anisotropic mechanical properties of silicon surfaces, the grooves formed on the “softest” Si(100) surface were the highest. On the contrary, the grooves on the “hardest” Si(111) surface indicated the lowest under the same loading conditions. However, the tribochemical removal of silicon by SiO_2_ tip (shown in Fig. 1) showed a completely different trend from the mechanical removal of silicon by diamond tip. Therefore, the anisotropic tribochemical wear of Si/SiO_2_ pair was not dependent on the crystallography-dependent surface mechanical properties of silicon surfaces.

## Discussions

### Microstructure analysis of tribochemical wear scars by XTEM

During wear tests of silicon surfaces by SiO_2_ spherical tip, the maximum normal load was 3.0 μN. Based on DMT contact theory^31^, maximum contact pressure *P*_c_ in the Si/SiO_2_ contact area was estimated (see Figure S2 in Supplementary Information). These contact pressures are much lower than the critical pressures for initial yield of silicon surfaces. Therefore, during the wear tests, the contact between silicon substrates and SiO_2_ tip must be elastic. In order to verify it, the nanowear tests of Si/SiO_2_ pair were performed at the same loading conditions in vacuum. As shown in Figure S3 of Supplementary Information, different from the severe tribochemical wear in humid air and water, tribochemical wear was suppressed in vacuum and no discernible material loss was observed on three crystal planes of silicon. Only a slight protruding structure formed in the contact area, which resulted from the amorphization of crystal silicon^24^. The preceding results demonstrated that tribochemical reaction dominated the material loss of silicon surface, and tribochemical wear was highly crystallography-dependent.

**Figure 3:**
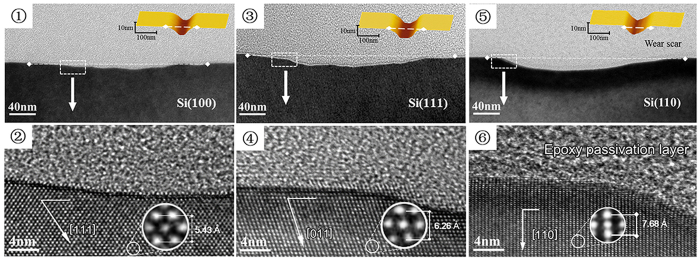
High-resolution TEM images of the wear tracks on Si(100), Si(111) and Si(110) samples after rubbed by SiO_2_ tip. Nanowear tests were performed under the condition of *F*_n_ = 3 μN in ultrapure water. HRTEM images and representative lattice resolved images marked with a frame (white dotted line) show ~4.6 nm, 6.7 nm and 7.5 nm deep wear scars formed on Si(100) (**①②**), Si(111) **(③④)** and Si(110) **(⑤⑥)** surface, respectively. Inset AFM images show the corresponding three-dimensional topography of the wear scars.

To understand further the anisotropic tribochemical wear of Si/SiO_2_ pair, the atomic characterization of the worn area on the Si(100), Si(110) and Si(111) surfaces were detected by cross-sectional transmission electron microscope (XTEM). Compared with the serious dislocation and deformation (Figure S4 of Supplementary Information), no crystal lattice distortion and dislocation were detected beneath the wear area of Si(100), Si(110) and Si(111) surfaces, as shown in Fig. 3. Under the given loading conditions, the pressed depths on all silicon surfaces were less than 0.2 nm. Thus, tribochemical reaction usually occurred on 1–2 atomic layers at the outermost surface of the silicon substrate, and the atomic layers in the subsurface remained intact. Based on the previous studies, the tribochemical wear in Si/SiO_2_ pair could be explained with a model involving the formation of “Si-O-Si” bonding bridges across the sliding interface and the rupture of Si-Si network^19^,^26^,^27^,^32^. The strained Si-O-Si networks in the contact area could facilitate the hydrolysis reaction to break the Si-O bonds, then the surface Si atoms could be removed as Si_m_(OH)_n_ debris. Moreover, the cross section of Si(100), Si(110) and Si(111) shows different atomic lattice arrangement. Given that the invisible atomic was located at the interior of the cubic diamond structure, the monoatomic layer shown in TEM images was indicated as a double layer in the actual silicon atomic structure. The thickness of double layers were estimated to be 2.715 Å for Si(100), 3.84 Å for Si(110) and 3.31 Å for Si(111), which were consistent with the theoretical values. Therefore, both the contact mechanism analysis and XTEM detection on the wear area confirmed the tribochemical wear mechanism of Si/SiO_2_ pair.

### Crystallography-induced anisotropy in tribochemical wear of Si/SiO_2_ pair

**Figure 4:**
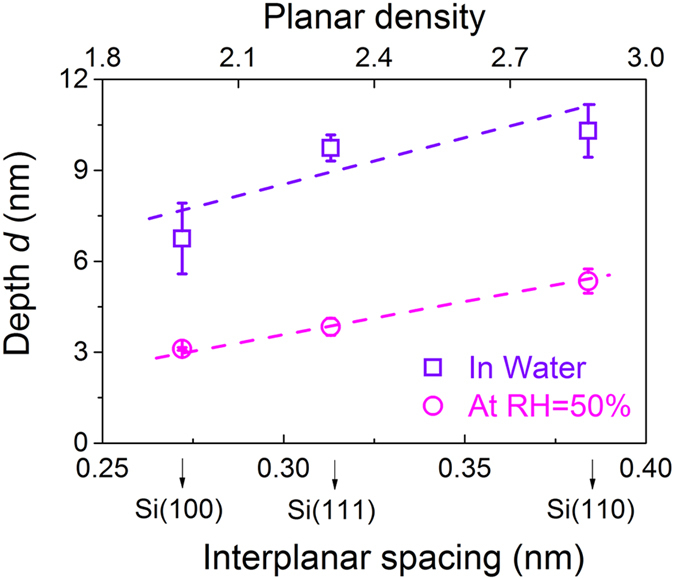
Correlation between interplanar spacing/atomic planar density and the wear depth of silicon samples in water and at 50% RH. The applied normal load was 3 μN. The interplanar spacing referred to the spacing thickness of double atomic layer.

Due to the surface effect, the surface density and interplanar spacing of substrate may to a certain extent, affect the tribochemical removal of silicon surface. Figure 4 shows the correlation between wear depth *d* and interplanar spacing/planar density of three silicon samples in water and at 50% RH. The results indicated that the silicon surface with larger atomic planar density and interplanar spacing suffered more severe damage. When the atomic planar density increased from 2.0 to 2.83 (1/a^2^) and interplanar spacing increased from 2.72 Å to 3.84 Å, the depths of wear scars increased from 6.5 nm to 10.5 nm in water and 3 nm to 5 nm at 50% RH.

Previous studies showed that the bond energy has an significant impact on the material removal behaviors^15^,^19^,^22^,^27^,^32^. Barnette *et al*. reported that the tribochemical removal on silicon surface can be effectively prevented by increasing the energy barrier of Si-O-Si bond dissociation with the ethanol adsorption^32^. On the contrary, water adsorption will reduce the activation energy of Si-Si network and facilitate the tribochemical wear of silicon^19^,^27^. It is also well known that the atomic bonds with larger atomic spacing could rupture more easily due to the lower atomic bond energy^33^. Based on the above tribochemical wear mechanism, the formation of “Si-O-Si” bonding bridges across the sliding interface and the rupture of Si-Si network were the most critical steps in tribochemical removal processes. Therefore, planar density and interplanar spacing of the Si substrate had a strong effect on the rate of tribochemical removal of the Si/SiO_2_ interface. On one hand, higher planar density provided more Si atoms to participate in the chemical reaction, which promoted the probability of the formation of Si-O-Si bonds in the contact area. On the other hand, when the Si-Si network was sheared and strained, larger interplanar spacing with weaker bonding energy was conducive to the formation and expansion of defects in the subsurface atomic layer. This effect facilitated hydrolysis reaction and removal rate of the silicon surface. As a result, the tribochemical removal rate of silicon was significantly improved by the combined effect of higher efficiency in both formation and rupture of Si-O-Si bonding bridges. Combined with the mechanical wear results, the anisotropic tribochemical removal was not dependent on the crystallography-dependent surface mechanical properties (i.e., hardness and elastic modulus) of silicon surfaces, but was mainly attributed to various atomic planar density and interplanar spacing in different crystal planes.

## Conclusion

The effect of crystal plane orientation on the tribochemical material removal of Si/SiO_2_ pair was investigated by AFM as a function of applied normal load and relative humidity. The experimental results indicated that crystal plane orientation had a prominent effect on water-involved tribochemical wear on the silicon surface. Combined with the mechanical wear results, such anisotropic tribochemical removal of silicon was not dependent on the crystallography-dependent surface mechanical properties (i.e., hardness and elastic modulus), but was mainly attributed to various atomic planar density and interplanar spacing in different crystal planes. Compared Si(100) with Si(110), the atomic planar density increased from 2.0 to 2.83 (1/a^2^) and interplanar spacing increased from 2.72 Å to 3.84 Å. As a result, the depths of wear scars increased from 6.5 nm to 10.5 nm in water and 3 nm to 5 nm at 50% RH. Phenomenological results speculated that higher planar density and larger interplanar spacing were conducive to the formation of Si-O-Si bonds between the contact interface and the rupture of Si-Si network in the subsurface of silicon. The investigation may help us understand the material removal mechanism of silicon and provides useful knowledge for chemical mechanical polishing of silicon wafers.

## Experimental

### Materials

**Figure 5:**
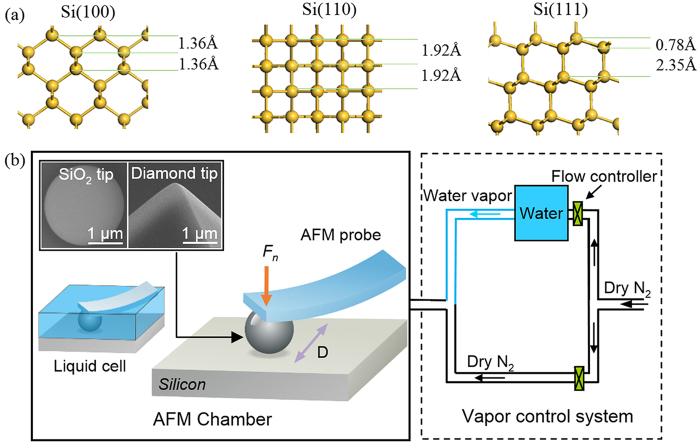
The schematic illustration showing the atomic structure of silicon materials and nanowear test details. **(a)** The configuration of Si(100), Si(110) and Si(111) samples. **(b)** The upper AFM tips moved horizontally on the silicon samples with displacement amplitude *D* of 500 nm and normal loads of 0.5 ~ 3 μN. The inset SEM images show the SiO_2_ tip with *R* of 1 μm and the diamond tip with *R* of 0.25 μm. The relative humidity was controlled from 0% to 80%.

The p-type Si(100), Si(110) and Si(111) wafers doped with trace boron were purchased from MEMC Electronic Materials, Inc., USA. The error of crystal plane orientation angle was less than 0.2%. Figure 5a shows the configuration of three types of monocrystalline silicon. The interplanar spacing of Si(100) and Si(110) are 1.36 Å and 1.92 Å, respectively. However, crystal plane (111) is the cleavage plane of silicon, which has long interplanar spacing (2.35 Å) and short interplanar spacing (0.78 Å). As a typical anisotropic material, monocrystalline silicon presents different elastic modulus on various crystal planes, i.e. 130 GPa on Si(100), 169 GPa on Si(110) and 188 GPa on Si(111), respectively, as shown in Table 1 24.

By using an AFM (SPI3800N, Seiko Instruments Inc., Tokyo, Japan), the root-mean-surface roughness of three kinds of silicon wafers was measured as no more than 0.5 nm over an area of 5 μm × 5 μm. To eliminate the effect of the native oxide layer (thickness ≈ 0.5 nm) on the material removal of the silicon substrate, silicon wafers were dipped in 5 wt.% hydrofluoric acid (HF) solution for 2 min to remove the oxide layer^34^. The samples were then ultrasonically cleaned with acetone, ethanol and deionized water for 3 min in sequence to remove surface contamination^35^.

### Methods

The nanoindentation tests on silicon wafers were conducted by a triboindenter (TI750, Hysitron Inc., MN, USA). During the tests, a spherical diamond indenter with nominal curvature radius *R* of approximately 1.5 μm was used. Nanowear tests on silicon wafers at various RHs were performed by the AFM equipped with an ambient chamber, as shown in Fig. 5b. Two kinds of tips were used in the wear tests, namely, a spherical SiO_2_ tip with a nominal radius *R* of about 1 μm (Novascan Technologies, USA) and a cubic corner diamond tip with *R* of about 0.25 μm (Micro Star Technologies, TX, USA). Through a standard cantilever with a spring constant of approximately 3.438 N/m (CFFC-NOBO, Veeco, USA), the cantilever spring constants of SiO_2_ and diamond tips were calibrated to be ~18.3 N/m and ~193 N/m, respectively^36^. During wear tests, the applied normal load *F*_n_ ranged from 0.5 μN to 3 μN. If not specially mentioned, the sliding speed *v* was 2 μm/s, the number of sliding cycles *N* was 100, the displacement amplitude *D* was 500 nm, and the temperature was controlled at 23 ± 2 °C. By using a humility-controlled chamber of AFM, RH varied between 0% and 80% with an error of ±2%. To remove residual moisture on silicon wafers, the chamber was pre-vacuumed by a pump and then refilled with the mixture of dry and humid air. Through a self-developed liquid cell, the wear tests could be realized in deionized water with a conductivity of 0.5 μs/cm obtained from laboratory water purification system (Master-S15, Hi-tech, China). Further details on the system composition and experimental process could be found in the previous literatures^15^,^27^.

### AFM and XTEM characterization

All AFM images were scanned by using silicon nitride probes (MLCT, Veeco, USA.) with a curvature radius of approximately 20 nm and spring constant of around 0.1 N/m^37^. Before imaging, the AFM chamber was pumped into ~5 × 10^−4^ torr vacuum to avoid the influence of adsorbed water film on samples. The atomic structure on the worn area of silicon samples with different crystal plane orientations was detected using XTEM (Tecnai G2 F20, FEI, USA). An epoxy polymer passivation layer was deposited on the silicon surface to protect the wear area from the damage by subsequent focused ion beam milling (NanoLab 400, FEI, USA).

## Additional Information

**How to cite this article:** Xiao, C. *et al*. Effect of crystal plane orientation on tribochemical removal of monocrystalline silicon. *Sci. Rep.* **7**, 40750; doi: 10.1038/srep40750 (2017).

**Publisher's note:** Springer Nature remains neutral with regard to jurisdictional claims in published maps and institutional affiliations.

## Supplementary Material

Supplementary Information

## Figures and Tables

**Table 1:** Comparison of the mechanical properties and atomic structure of three silicon samples.

Sample	Si(100)	Si(110)	Si(111)
Elastic modulus (GPa)	130	169	188
Hardness (GPa)	11.3	13.0	13.2
Atomic planar density (1/a^2^)	2.0	2.83	2.3
Double layer thickness (Å)	2.72	3.84	3.13

(a is the lattice constant of silicon, 0.543 nm).
