# Supplementary material for: Effect of crystal plane orientation on tribochemical removal of monocrystalline silicon
Source: Sci Rep. 2017 Jan 13;7:40750. doi: 10.1038/srep40750 (PMC5233974; doi:10.1038/srep40750)
Supplement: Supplementary Information [file srep40750-s1.pdf]

Supplementary Information

Effect of crystal plane orientation on tribochemical removal of monocrystalline silicon

Chen Xiao[1], Jian Guo[1,2], Peng Zhang[1], Cheng Chen[1], Lei Chen[1], Linmao Qian[1]*

[1]Tribology Research Institute, National Traction Power Laboratory, Southwest Jiaotong University, Chengdu 610031, Sichuan Province, P. R. China

[2]School of Mechatronics Engineering, University of Electronic Science and Technology of China, Chengdu 611731, Sichuan Province, P. R. China

[*] Corresponding Author: linmao@swjtu.edu.cn, Tel.: +86 28 87600687 and Fax: +86 28 87603142

1. Effect of crystal plane orientation on the friction of Si/SiO_2 pair

Figure S1 shows the friction coefficient of the silicon samples with different crystal plane orientations rubbed by SiO_2 tip in humid air and in water. The friction force was calibrated by a modified wedge method using a silicon grating with a wedge angle of 54°44′ (TGF11, Mikro Masch, Germay). There is almost no difference between the friction coefficient of silicon samples with different crystal plane orientations either in humid air or in water. Therefore, the anisotropic tribochemical removal of silicon was not attributed to the friction behavior.

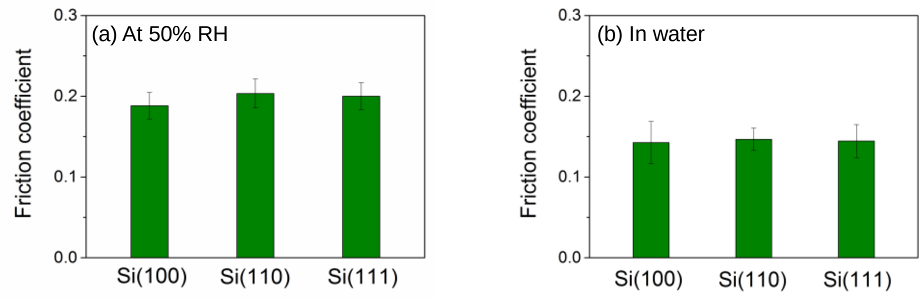

Figure S1. Comparison of the friction coefficient of the silicon samples with different crystal plane orientations rubbed by SiO_2 tip in humid air (a) and in water (b).

2. Effect of crystal plane orientation on the contact pressure of Si/SiO_2 pair

During the wear tests of silicon surfaces by SiO_2 spherical tip, the maximum applied normal load was 3.0 μN. Based on the DMT contact mechanics theory[1], the maximum contact pressure P_c in the Si/SiO_2 contact area was estimated, as shown in Figure S2. The critical contact pressures (P_y) of three silicon samples were referred the scratch results reported by Yu et al., where the critical contact pressure that led to the appearance of groove was approximately 11.3 GPa on Si(100), 13.4 GPa on Si(110) and 14.2 GPa on Si(111)[2]. These contact pressures were much lower than the critical pressures for initial yield of silicon surfaces. Therefore, during the wear tests, the contact between silicon substrates and the SiO_2 tip must be elastic. The material removal on silicon surface by rubbed with SiO_2 tip must be due to chemical reaction or facilitated by mechanical shear.

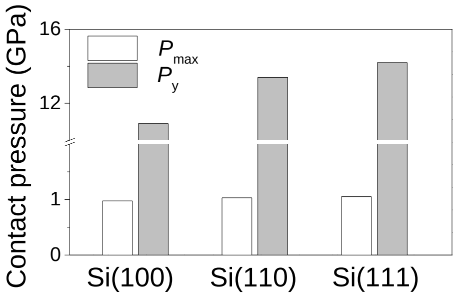

Figure S2. Comparison of the maximum contact pressure (P_{max}) with the critical contact pressure (P_y) of Si/SiO_2 pairs. P_{max} was calculated by DMT model between SiO_2 microspheres with the radius $R \approx 1$ μm and various silicon substrates with different crystal planes.

3. Effect of crystal plane orientation on nanowear of Si/SiO_2 pair in vacuum and water

Figure S3 shows the nanowear test results of Si/SiO_2 pair in vacuum and water. Different from the severe material removal in humid air and water, the tribochemical wear was suppressed in vacuum and no discernible material loss was observed on three crystal planes of silicon. Only a slight protruding structure about 0.5 nm was formed in the contact area, which was resulted from the amorphization of crystal silicon[3]. In this case, mechanical wear dominated the damage on the

silicon surfaces, it was also verified that the mechanical interaction could cause only a mild mechanical damage (hillock formation) under given loading conditions and the difference in material removal of various Si/SiO_2 pairs was mainly attributed to the crystallography-induced anisotropy in tribochemical reaction[4].

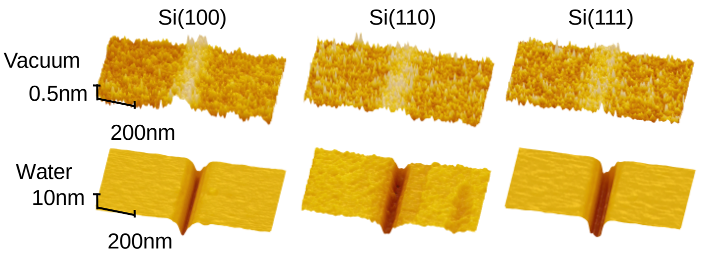

Figure S3. AFM images of wear scars on three silicon surfaces rubbed by SiO_2 microsphere in vacuum and water. The applied normal load was 3 μN. The height full-scales for wear scars in vacuum were 0.5 nm and that in water were 10 nm.

4. XTEM analysis on cross-section of Si(100) groove rubbed by diamond tip

As a comparison, the microstructure of groove with about 10 nm in depth on Si(100) produced by diamond tip was analyzed by the XTEM. As shown in Figure S4, a thick amorphous silicon layer with thickness of ~150 nm was observed in the HRTEM image. The thickness of the mechanically interacting layer was much larger than the depth of groove. Selected-area diffraction (SAD) pattern A indicated that an obvious amorphous silicon layer was formed under the groove. Beneath the amorphous layer a deformed zone with large-area distorted crystal matrix was displayed. Detail detection in Figure S4(C) shows the distinct intersectional slip bands with dislocations. As the slip lines extended into the bulk which defined the characteristic V-shape of the dislocation distribution. As the same as Si(100), such slip bands in deformation zone of Si(110) surface were also parallel to the {111} planes[5]. Unlike the observation in Si(111) by Haq and Munroe, who reported that slip bands were found both on {111} and {311} planes because of the special atomic structure[6]. Meanwhile, the density of defects and distribution on Si(111) and

Si(110) was significantly lower than that on Si(100)[5, 6].

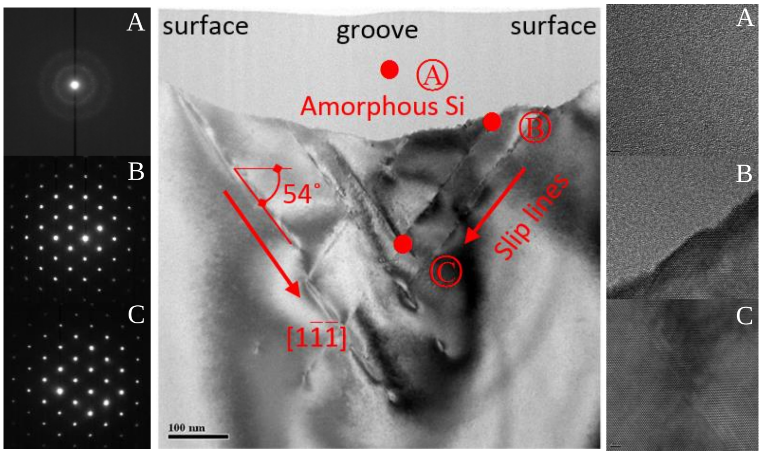

Figure S4. XTEM image showing the cross-sectional structure of a typical groove on Si(100) surface rubbed by diamond tip. The left pictures show the selected-area diffraction (SAD) patterns from area. The middle picture showing the XTEM image of whole scratching area. The right pictures showing the detail images in the characteristic regions marked in the middle picture. The applied normal load F_n was 50 μN, sliding cycle N was 100 and velocity v was 20 μm/s.

References

1. Xu, D., Liechti, K. M. & Ravi-Chandar, K. On the modified Tabor parameter for the JKR-DMT transition in the presence of a liquid meniscus. *J. Colloid Interf. Sci.* **315**, 772-785 (2007).

2 Yu, B. J. & Qian, L. M. Effect of crystal plane orientation on the friction-induced nanofabrication on monocrystalline silicon. *Nanoscale Res. Lett.* **8**, 1-8 (2013).

3 Yu, B. J. et al. Towards a deeper understanding of the formation of friction-induced hillocks on monocrystalline silicon. *J. Phys. D: Appl. Phys.* **45**, 145301 (2012).

4 Wang, X. D. *et al.* Humidity dependence of tribochemical wear of monocrystalline silicon. *ACS Appl. Mater. Interfaces* **7**, 14785-14792 (2015).

5. Jian, S. R., Chen, G. J. & Juang, J. Y. Nanoindentation-induced phase transformation in (110)-oriented Si single-crystals. *Curr. Opin. Solid State Mater. Sci.* **14**, 69-74 (2010).

6 Haq, A. J. & Munroe, P. R. Phase transformations in (111) Si after spherical indentation. *J. Mater. Res.* **24**, 1967-1975 (2011).
